# Case report: Germline RECQL mutation potentially involved in hereditary predisposition to acute leukemia

**DOI:** 10.3389/fonc.2023.1066083

**Published:** 2023-03-14

**Authors:** Wei Yuan, Zhen Shang, Kefeng Shen, Qiuxia Yu, Qiuxia Lv, Yang Cao, Jue Wang, Yi Yang

**Affiliations:** ^1^ Department and Institute of Infectious Disease, Tongji Hospital of Tongji Medical College, Huazhong University of Science and Technology, Wuhan, China; ^2^ Department of Hematology, Tongji Hospital of Tongji Medical College, Huazhong University of Science and Technology, Wuhan, China; ^3^ Immunotherapy Research Center for Hematologic Diseases of Hubei Province, Tongji Hospital of Tongji Medical College, Huazhong University of Science and Technology, Wuhan, China; ^4^ Department of geriatrics, Tongji Hospital, Tongji Medical College, Huazhong University of Science and Technology, Wuhan, China

**Keywords:** case report, acute leukemia, germline mutation, hereditary predisposition, fusion gene

## Abstract

The pathogenesis of acute leukemia is still complex and vague. Most types of acute leukemia are related to somatic gene mutations, and familial incidence is rare. Here we report a case of familial leukemia. The proband presented to our hospital with vaginal bleeding and disseminated intravascular coagulation at the age of 42 and was diagnosed with acute promyelocytic leukemia with typical *PML-RARα* fusion gene caused by t(15;17)(q24;q21) translocation. By taking the history, we found that the patient’s second daughter had been diagnosed with B-cell acute leukemia with *ETV6-RUNX1* fusion gene at age 6. Then we performed whole exome sequencing in peripheral blood mononuclear cells from these two patients at remission status and identified 8 shared germline gene mutations. Using functional annotation and Sanger sequencing validation, we finally focused on a single nucleotide variant in RecQ like helicase (*RECQL*), rs146924988, which was negative in the proband’s healthy eldest daughter. This gene variant potentially led to a relative lack of RECQL protein, disordered DNA repair and chromatin rearrangement, which may mediate the occurrence of fusion genes, as driving factors for leukemia. This study identified a novel possible leukemia-related germline gene variant and provided a new understanding for the screening and pathogenesis of hereditary predisposition syndromes.

## Introduction

In addition to the somatic changes related to diagnosis and prognosis in leukemia cells, many germline mutations have been discovered in hematopoietic malignancies in recent years, namely hereditary predisposition (1, 2). The ‘Myeloid Neoplasms with Germline Predisposition’ category has been included in the revised fourth edition of the World Health Organization (WHO) classification of tumors of the hematopoietic and lymphoid tissues (3), which further emphasizes the importance of constitutional mutation in the occurrence and development of hematological neoplasms. Fusion genes are hybrid genes formed by the fusion of two previously separated genes, which are resulting from chromosomal rearrangement, including translocation, inversion, deletion, or tandem duplication (4). Fusion genes are molecular biological characteristics of many leukemia types and have been successfully used as diagnostic markers and therapeutic targets (5). However, its relationship with hereditary predisposition is still unclear.

Here, we report a case of familial acute leukemia with fusion genes and germline RecQ Like Helicase (*RECQL*) mutation. The proband was a 42-year-old female patient with acute promyelocytic leukemia (APL) and *PML-RARα* fusion gene. She has two daughters, and the younger one developed B-cell precursor acute lymphoblastic leukemia (BCP-ALL) with *ETV6-RUNX1* fusion gene at age 6. As of the date of submission, the eldest daughter is still in good health. Comprehensive genetic testing showed that a single nucleotide variant (SNV) in *RECQL*, rs146924988, was potentially involved in this familial case with acute leukemia.

## Case presentation

### Case 1 (mother)

A previously healthy 42-year-old woman presented to the emergency room due to massive vaginal bleeding in November 2021. Blood routine test showed an abnormal increase of white blood cells (WBC) of 32.83×10^9/L and monocytes of 20.19×10^9/L, with hemoglobin of 100.0g/L and decreased platelet count of 43.0×10^9/L. Coagulation function test indicated a prolonged prothrombin time of 26.8s, decreased fibrinogen level of 0.93g/L and elevated fibrinogen degradation products of 89.7μg/mL and D-Dimer greater than 21μg/mL FEU, suggesting a disseminated intravascular coagulation. An intramural uterine fibroid (approximately 1.8cm*1.3cm) on the anterior wall was found by transabdominal pelvic ultrasonography. Peripheral blood cells smear showed that granulocytes and promyelocytes accounted for 92.00% and 88.00% of the total number of nucleated cells, respectively.

The patient was then admitted to the hematology department for further diagnosis and treatment. Morphological examination of bone marrow (BM) aspirates revealed an abnormal myeloid development with an increased percentage of promyelocytes (approximately 92.00%). BM biopsy analysis revealed marked hyperactive hyperplasia and promyelocytic dysplasia and cytosis (about 90%), and the immunohistochemistry stains for MPO and CD117 were both positive. Flow cytometric immunotyping of BM aspirates showed that abnormal myeloblasts expressed CD117, CD45, cMPO, CD64, CD9, CD33bri, CD13 and CD38, accounting for 83.3% of the total nucleated cells. A few abnormal myeloblasts expressed CD34 (approximately 9.9%), CD11c and CD11b. These abnormal cells were large and had side scatter (SSC) values similar to those of granulocytes. Cytogenetic analysis showed an abnormal karyotype of 46, XX, t(15;17)(q24;q21), del (6)(q22;q32). Molecular genetic analysis detected a typical S-type *PML-RARα* fusion gene transcript caused by t(15;17)(q24;q21) translocation (approximately 60.30%). Based on these results, this patient was diagnosed as APL (7). After being classified as high-risk group, this patient received all-trans retinoic acid (ATRA, 25 mg·m^-2^·d^-1^, d1-d3^)^ combined with arsenic trioxide (0.16 mg·kg^-1^·d^-1^, d1-d3) induction chemotherapy, and continued with three courses of IA (idarubicin 8 mg·m^-2^·d^-1^, d1-d3; cytarabine 100 mg·m^-2^·d^-1^, d1-d5) consolidation regimen (6). Six times of prophylactic intrathecal chemotherapy were administered during this process to prevent central nervous system relapse. And then ATRA combined with compound Huangdai tablet (CHDT) was used as maintenance chemotherapy. To this date, the patient was still undergoing maintenance therapy and staying in complete remission at the cytological and molecular levels both in bone marrow and cerebrospinal fluid, with no signs or symptoms of APL recurrence.

### Case 2 (daughter)

**Figure 1:**
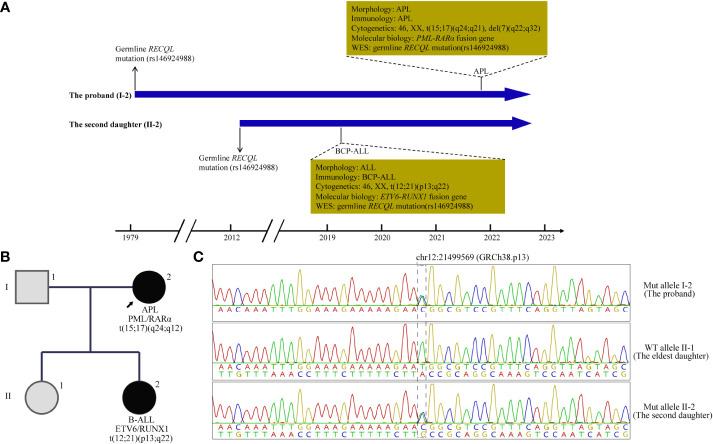
A brief summary of medical history and pedigree for the *RECQL* mutation carriers. **(A)** Timeline of clinical events in 2 patients with acute leukemia. **(B)** The Pedigree and germline and tumor chromatopherogram confirmed the presence of single nucleotide variation in *RECQL* (rs146924988) in 2 patients with acute leukemia in this family, while it was negative in healthy members. **(C)** Sanger sequencing of nasopharyngeal swab specimens was used to verify *RECQL* mutation (rs146924988) in the proband and her two daughters. APL, acute promyelocytic leukemia; WES, whole exome sequencing; BCP-ALL, B-cell precursor acute lymphoblastic leukemia; Mut, mutant; WT, wide type.

During medical history collection of case 1 (mother), it was found that a 6-year-old girl, the younger one of the patient’s two daughters, was diagnosed with BCP-ALL (low-risk group) in March 2019, just two years before the occurrence of her mother’s APL (**Figure 1A**). At the diagnosis, the blast cells in bone marrow were CD10, CD19 and terminal deoxynucleotidyl transferase (TdT) positive detected by fluorescence activated cell sorting (FACS), and with *ETV6-RUNX1* fusion gene detected by RT-PCR and t(12;21)(p13;q22) translocation by florescence *in situ* hybridization (FISH). After receiving a high-dose chemotherapy regimen according to the CCCG-ALL-2015 protocol (8), the daughter achieved complete remission. Up to this date, the daughter still underwent oral maintenance therapy with mercaptopurine (6-MP) and methotrexate (MTX) and remained in minimum residual disease (MRD) negative remission at cellular and molecular (*ETV6-RUNX1*) levels.

To explore the possible hereditary predisposition in this case, we performed whole exome sequencing (WES) in peripheral blood mononuclear cells (PBMCs) from these two patients at remission status with informed consents. The genomic DNA of each sample was sheared with Biorupter (Diagenode, Belgium) and 150 ~ 200bp fragments were obtained. AIExomeV1 Enrichment Kit (iGeneTech, Beijing, China) and Illumina platform (Illumina, San Diego, CA) were applied to capture and sequence the whole exons. Then clean reads were mapped to the reference genome GRCh37 by using Burrow-Wheeler Aligner. After removing low quality reads and duplications, SNV and insertion-deletion (InDel) were called and annotated by using Genome Analysis Toolkit (GATK). Finally, a total of 8 germline gene mutations shared by both patients were identified (**Table 1**), including 2 InDel and 6 SNV, in which an SNV in *RECQL* (rs146924988) was noticed by functional annotation. Sanger sequencing of nasopharyngeal swab specimens was used to verify WES results, and to screen the other healthy child of the proband without *RECQL* mutation detected. The pedigree for rs146924988 and leukemia was shown in **Figures 1B, C**.

**Table 1:** Summary of shared germline gene mutations at remission status.

Gene	Vcf_mut	AAChange.HGVS	FRE (case1/case2)	Mut_type	avsnp150
***RECQL***	chr12:21652503:A/G	c.2T>C:p.0?	45.1%/42.9%	SNP	rs146924988
*AK2*	chr1:33478892:G/A	c.586C>T:p.(Arg196Trp)	50.5%/49.6%	SNP	rs370429097
*C7*	chr5:40972681:C/T	c.2059C>T:p.(Arg687Cys)	47.8%/45.9%	SNP	rs117487879
*CCDC40*	chr17:78069197:G/A	c.2968G>A:p.(Asp990Asn)	50%/50.5%	SNP	rs200958035
*NCF4*	chr22:37273779:G/T	c.934G>T:p.(Gly312Cys)	46.9%/39.0%	SNP	rs199618052
*KCNQ4*	chr1:41284190:C/G	c.546C>G:p.(Phe182Leu)	52.7%/41.7%	SNP	rs80358273
*GJB2*	chr13:20763485:AG/A	c.235del:p.(Leu79Cysfs*3)	47.4%/54.9%	InDel	rs80338943
*USH2A*	chr1:216595579:G/GA	c.99_100insT:p.(Arg34Serfs*41)	49.0%/41.1%	InDel	rs141672841

Mut, mutation; FRE, frequency; InDel, insertion-deletion; SNP, single nucleotide polymorphism.

### Patient perspective

I have an entire family, including a husband and two daughters. I led a busy and happy life, struggling to make ends meet, until my little daughter and I were diagnosed with leukemia in turn. Before I came to Tongji Hospital, I thought my life was coming to an end. Fortunately, after receiving systematic treatment, my daughter and I were cured and there is no sign of recurrence. Our life and work are gradually returning to normal.

## Discussion

With the development of genomic sequencing methods, many germline mutations have been found in cancer patients and their immediate relatives. Germline mutations associated with the hereditary predisposition to cancers were highlighted for their clinical relevance and diagnostic values, which were also known as hereditary predisposition syndromes (HPS) (9). For hematological malignancies, hereditary predisposition was first identified in chronic lymphocytic leukemia (CLL) and acute myeloid leukemia (AML), which has led to the identification of several germline mutations, such as *RUNX1, CEBPA, GATA2, ANKRD26, DDX41* and *ETV6* mutations. Furthermore, genetic susceptibility was indicated in ALL and multiple myeloma, with constitutional mutations in genes such as *IKZF1, SH2B3, PAX5* (ALL) and *KDM1A/LSD1* (multiple myeloma). Monogenic genomic alterations have also been found to be involved in inborn errors of the immune system and confer a predisposition to hematological malignancies such as ataxia telangiectasia, Nijmegen breakage syndrome, Bloom’s syndrome, xeroderma pigmentosum, constitutional mismatch repair deficiency, Fanconi anemia, and telomere syndromes (2, 10–12). Interestingly, previously identified inherited cancer susceptibility syndromes also increase the possibility to develop hematological malignancies. For example, patients with Diamond-Blackfan anemia, severe congenital neutropenia, or familial thrombocytopenia have a significantly increased incidence of myeloid neoplasms (10). Up to 62% of children with lymphoproliferative diseases have primary immune deficiency disease (PID) (13). Compared with the general population, people with PID have a 1.5 times higher risk of developing cancer (14). At present, HPS-related genes or pathways involve transcription factors, RNA function, DNA methylation, ribosome assembly, histone modification, DNA unwinding and DNA repair (9). Other germline mutations in key transcription and translation processes and signal transduction pathways, such as the RAS pathway, cell proliferation, apoptosis, and tumor suppressor genes (e.g., *TP53*) were also considered as possible underlying predispositions.

**Figure 2:**
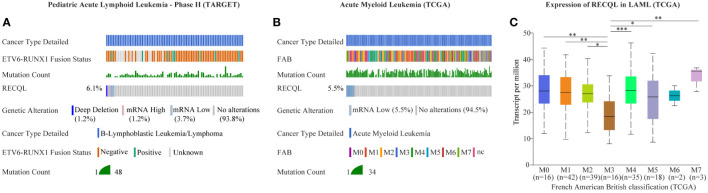
Mutation and expression of *RECQL* potentially involved in acute leukemia. The alteration frequency in *RECQL* in acute leukemia was analyzed by using the cBioPortal database, including data of AML from TCGA database and data of BCP-ALL from TARGET database. Samples with complete data of gene mutation, CNA and mRNA expression (log RNA Seq V2 RSEM) were retained after screening. **(A)** The alteration frequency in *RECQL* in B-ALL patients (Pediatric Acute Lymphoid Leukemia - Phase II, TARGET). **(B)** The alteration frequency in *RECQL* in AML patients (TCGA-LAML). **(C)** Comparison of *RECQL* gene expression in AML patients with different French American British classification. AML, Acute Myeloid Leukemia; TCGA, The Cancer Genome Atlas; B-ALL, acute B lymphoblastic leukemia; CNA, copy-number alterations; RSEM, RNA-Seq by Expectation Maximization; TARGET, therapeutically applicable research to generate effective treatments. Statistically significant differences are indicated as follows: *p < 0.05, **p < 0.01, ***p < 0.001.

In this case, the proband and her second daughter both suffered from acute leukemia at different times in their lives. Considering the incidence of acute leukemia, we speculated that there might be HPS in this case. Through WES sequencing and bioinformatics analysis, we finally focused on rs146924988, an SNV in *RECQL*, and the mutation frequencies in the proband and her second daughter were 45.1% and 42.9%, respectively. Further Sanger sequencing showed no such mutation of *RECQL* in the healthy child of the proband, which indicated that rs146924988 might play a role in this case of familial leukemia. The proband of this case was an APL patient, carrying a typical *PML-RARα* fusion gene. The second daughter of the proband was a BCP-ALL patient, carrying the classical *ETV6-RUNX1* fusion gene. Considering the driving role of these two different fusion genes in the occurrences and development of corresponding leukemias (15, 16), it can be inferred that this *RECQL* gene variant (rs146924988) is potentially associated with the generation of acute leukemia. We evaluated the alteration frequency and expression levels of *RECQL* in acute leukemia from publicly available datasets using the cBioPortal database (17), including AML datasets from The Cancer Genome Atlas (TCGA) (18) and B-ALL datasets generated by the Therapeutically Applicable Research to Generate Effective Treatments (TARGET, https://ocg.cancer.gov/programs/target) initiative, phs000464. Samples with complete data of gene mutations, copy-number alterations (CNA) and mRNA expressions were retained after screening. The CNA frequency of *RECQL* was more than 5% both in ALL and AML patients, and the main variants types are deep deletion and shallow deletion that related to low gene expression (**Figures 2A, B**). Interestingly, the expression of *RECQL* in AML-M3 patients mostly having *PML-RARα* fusion gene, was significantly lower than that in other types of AML patients (**Figure 2C**). These findings highlighted the potential role of *RECQL* in leukemia.

**Figure 3:**
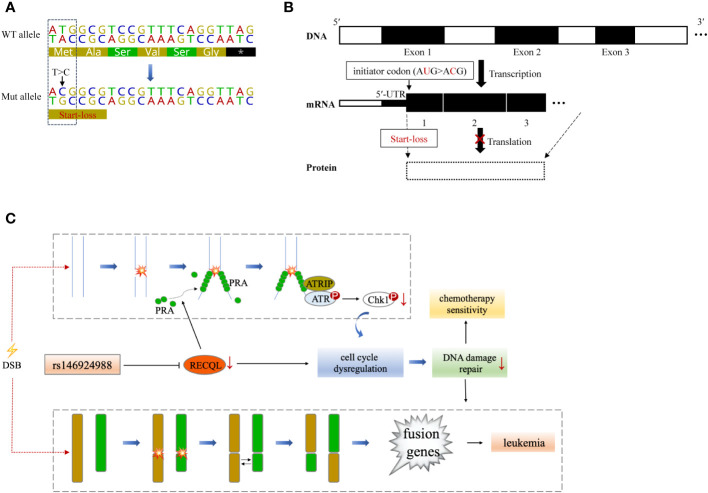
A theoretical model that *RECQL* mutation (rs146924988) acts as a potential mediator of hereditary leukemia. **(A)** Variant details of single nucleotide variation in *RECQL* (rs146924988). Dotted box indicates the corresponding DNA sequence of the initiator codon. **(B)** Hypothetical mechanism that *RECQL* mutation (rs146924988) affecting transcription and translation. The rs146924988 mutation is an initiator codon variant of *RECQL*, which may lead to a start-lost loss-of-function of RECQL. **(C)** A theoretical model that rs146924988 acts as a mediator of hereditary leukemia. The germline mutation (rs146924988) was proposed to mediate the occurrence of chromosome rearrangement and fusion genes, as well as affect chemotherapy sensitivity. WT, wide type; Mut, mutant; DSB, DNA double-strand break.

*RECQL* gene, as a member of the RecQ helicase family located on chromosome 12p12, encodes a DNA helicase with 649 residues. RECQL protein is a highly conserved protein and plays an important role in DNA repair, replication, recombination and transcription (19). The SNV (rs146924988) detected in this case is an initiator codon variant of *RECQL*, which may lead to start-lost loss-of-function of RECQL (20, 21) (**Figures 3A, B**). Prior to this report, the *RECQL* germline mutation has been found to be associated with hereditary breast cancer and hereditary ovarian cancer syndrome (22, 23), but its role in hematological malignancies has not been reported. RECQL protein is an ATPase and helicase that can bind and unlock the structural intermediate of DNA replication and repair. RECQL unlocks double-stranded DNA and catalyzes the migration of ATP-dependent branches on Holliday junctions and removable D-loop substrates. In addition to cleavage of DNA, RECQL promotes annealing of complementary single-stranded DNA in an ATP-independent manner (24). Moreover, RECQL can also interact with proteins that are involved in DNA replication, repair and mismatch repair, such as FEN1, RPA, PARP1, and MLH1 (25–27). Deletion of RECQL resulted in increased DNA damage accumulation, which indicated that RECQL was involved in the solution of replication fork stagnation. RECQL-deficient mice and cells exhibited frequent spontaneous chromosomal breakage and chromatid translocation (28). In a recent *in-vitro* experiment based on MDA-MB-231 cells, RECQL was considered to be involved in regulating cell stress response (29). It has been found to mediate the recruitment of replication protein A (RPA) in DNA damage sites and to promote the activation of the ATR-Chk1 pathway in response to the DNA damage caused by gemcitabine (29). ATR-Chk1 is a classic pathway of DNA damage response (DDR) induced by DNA double-strand breaks (DSBs), involving cell cycle checkpoint regulation and DNA repair (30). It can promote ubiquitination and proteasome degradation by phosphorylating CDC25 phosphatases (CDC25A, B, C), and induce cell cycle G1-S transition and G2-M transition (31–33). ATR-Chk1 dysfunction is associated with a large number of DNA damage and chromosomal rearrangements in Ataxia-telangiectasia (A-T) patients, especially in lymphocytes, which often leads to the production of fusion genes (34, 35).

Based on these previous findings, we hypothesize that the constitutional mutation in *RECQL* gene (rs146924988) may inhibit the translation of *RECQL* mRNA and lead to a relative lack of RECQL protein, which may further impair the ATR-Chk1 pathway and resulting in functional disorders in DNA replication and DSBs repair. It is conceivable that these functional disorders could ultimately mediate the occurrence of chromosome rearrangement and fusion genes, as driving factors for leukemia (**Figure 3C**). Admittedly, we have not obtained direct evidence that *RECQL* gene mutation (rs146924988) will lead to decreased *RECQL* expression level because this study is a case report in nature, and the biological process of such mutation potentially affecting DSBs repair and mediating the production of fusion genes needs to be verified in *in-vitro* experiments and larger clinical cohorts in the future. Moreover, there were 7 other shared germline mutations in this case of familial leukemia, which were related to genes *AK2*, *C7*, *CCDC40*, *NCF4*, *KCNQ4*, *GJB2* and *USH2A*. These genes were reported to be involved in ADP biosynthesis, complement activation, oxidation-reduction and other biological processes, and their functional annotations were shown in **Table S1**. By checking the literature and public data repository, there is no direct evidence that these mutations are associated with genomic instability or genetic predisposition to cancer, but their contributions to the onset of leukemia cannot be completely excluded and needs further evaluation. The loss of RECQL function may increase the probability of the somatic events, such as the occurrence of chromosome rearrangements, in positions given by chance and modulated by other factors, resulting in different genetic fusions genes. This partly explained how germline genetic variants in *RECQL* lead to different types of fusions gene in these 2 cases.

Early recognition of the hereditary predisposition of hematological malignancies is critical for timely diagnosis and individualized treatment. In this study, a potential HPS-related gene mutation was identified by WES sequencing, which highlights the clinical application of WES, especially in patients with early-onset leukemia or familial leukemia. In addition to diagnostic value, screening of the HPS-related gene mutations may provide prognosis evaluation reference for an optimal intervention approach. For example, previous studies have shown that *RECQL* gene defects not only cause genomic instability and chromatin recombination, but also make cells more sensitive to toxic genomic stress and cytotoxic therapy (29). Particularly, polymorphic variants in *RECQL* was found to be related to the overall survival rate of pancreatic cancer patients treated with gemcitabine (36). RECQL helicase has also been found to protect multiple myeloma cells from melphalan and bortezomib cytotoxicity (37). Therefore, the significance of *RECQL* mutation in the treatment of acute leukemia needs to be evaluated in future studies.

In this study, we performed WES sequencing in a familial leukemia case, and for the first time to our knowledge, found a germline *RECQL* mutation potentially involved in hereditary predisposition to acute leukemia. The hypothetical biological process in which *RECQL* gene mutation (rs146924988) affect DSBs repair and mediate the generation of fusion genes provides a new understanding of the pathogenesis of leukemia, and highlights the necessity for next-generation sequencing-based screening of genes involved in this process in potential HPS patients.

## Data availability statement

The datasets presented in this article are not readily available because of ethical/privacy restrictions. Requests to access the datasets should be directed to the corresponding author.

## Ethics statement

This study was approved by the Medical Ethics Committee of the Department of Hematology, Tongji Hospital, Tongji Medical College, Huazhong University of Science and Technology. Written informed consent to participate in this study was provided by the participants’ legal guardian/next of kin.

## Author contributions

All authors designed the study, interpreted the findings and revised the manuscript. WY, ZS, KS, QY, and QL carried out data management and statistical analysis and drafted the manuscript. YC and JW helped with English language editing and data management. YY performed project administration. All authors contributed to the article and approved the submitted version.
